# Osler’s nodes in infective endocarditis

**DOI:** 10.1016/j.idcr.2023.e01862

**Published:** 2023-07-28

**Authors:** Rolando A. Zamora Gonzalez, Lena J. Heung

**Affiliations:** a Division of Infectious Diseases, Department of Medicine, David Geffen School of Medicine at the University of California, Los Angeles, CA, USA; b Division of Infectious Diseases, Department of Medicine, Cedars-Sinai Medical Center, Los Angeles, CA, USA

**Keywords:** Osler's nodes, Aortic valve endocarditis, Staphylococcus aureus, MSSA

A 40-year-old male with obesity and type 2 diabetes mellitus presented to the emergency department with four days of fever, chills, night sweats, and left upper quadrant abdominal pain. He had a history of recurrent back abscesses, and two weeks prior to presentation, he had an abscess that spontaneously drained and resolved on its own. Physical exam on presentation was notable for fever (39 °C), tachycardia (130 beats per minute), a I/IV diastolic murmur at the lower left sternal border with no radiation, and violaceous, tender nodular lesions on his left thumb and thenar eminence (Fig. 1A), consistent with Osler’s nodes. Further workup revealed high-grade methicillin-sensitive *Staphylococcus aureus* bacteremia. An initial transthoracic echocardiogram was negative, but a subsequent transesophageal echocardiogram revealed aortic valve vegetations with severe regurgitation (Fig. 1B). Computed tomography (CT) of abdomen and pelvis revealed a splenic infarction (Fig. 1C) that correlated with the patient’s abdominal pain on presentation. He was evaluated by cardiothoracic surgery and recommended for valve replacement. On a pre-operative CT of the brain, he was found to have an asymptomatic infarction in the right occipital lobe (Fig. 1D). The patient underwent a mechanical aortic valve replacement and did well after completing a six-week course of intravenous antimicrobials (2 weeks of oxacillin followed by 4 weeks of cefazolin).

**Fig. 1:**
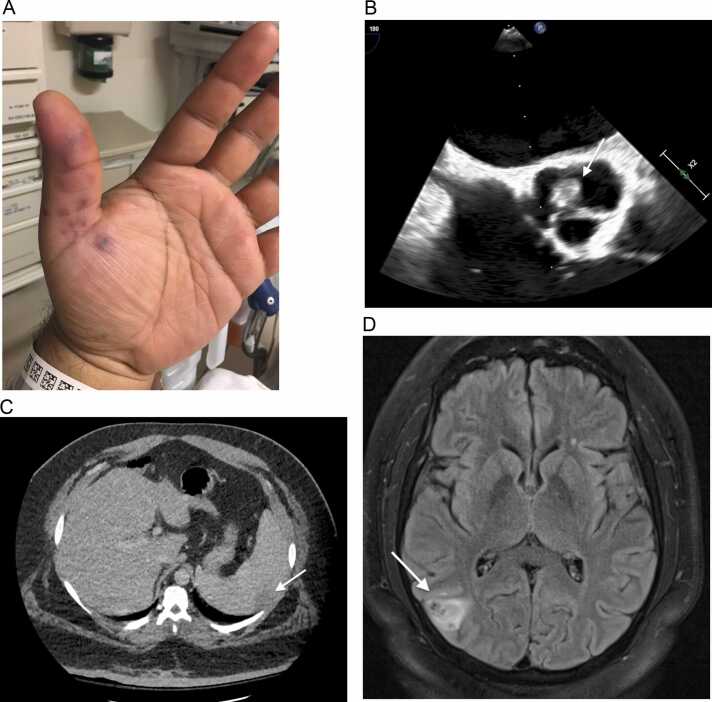
A. Osler’s Nodes in left thumb extending into palm. B. Transesophageal echocardiogram showing aortic valve vegetations. C. Wedge shaped area of decreased attenuation concerning for splenic infarction. D. Small acute to subacute infarction in the right occipital lobe, likely related to embolic focus. Small associated focal hemorrhage at this site is also seen.

Osler’s nodes are classically purple, painful cutaneous lesions on the hands or feet that are thought to be microembolic versus immunologic phenomena. They are an uncommon clinical finding but highly suggestive of left-sided infective endocarditis. Thus, identifying Osler’s nodes can be particularly helpful, as was highlighted in this case where an initial transthoracic echocardiogram was negative for vegetations. Timely diagnosis of infective endocarditis is critical given high morbidity and mortality without appropriate therapy.

## Ethical approval

Not needed for casereports at our institution.

## Consent

Consent was obtained from patient directly.

## Funding

None.

## CRediT authorship contribution statement

Both authors contributed equally in creating this submission.

## Declaration of Competing Interest

LJH reports research funding from the National Institutes of Health/National Institute of Allergy and Infectious Diseases for unrelated studies and an unpaid role as a member of the IDWeek Program Committee All authors declare no competing interests.

